# The social cost of gathering information for trust decisions

**DOI:** 10.1038/s41598-020-69766-6

**Published:** 2020-08-21

**Authors:** I. Ma, A. G. Sanfey, W. J. Ma

**Affiliations:** 1 Donders Institute, Radboud University, Nijmegen, The Netherlands; 2 Behavioural Science Institute, Radboud University, Nijmegen, The Netherlands; 3 New York University, New York, USA

**Keywords:** Human behaviour, Psychology

## Abstract

Trust decisions are inherently uncertain, as people usually have incomplete information about the trustworthiness of the other person prior to their decision to trust or not trust. Therefore, it is typically beneficial to gather information about a trustee’s past behaviour before deciding whether or not to trust them. However, elaborate inquiries about a trustee’s behaviour may change the trustee’s willingness to reciprocate, causing either a decrease due to the trustee’s negative impressions of the investor or an increase because the investor appears to be highly betrayal-averse to the trustee. In turn, such a change could cause the investor to gather less or more information, respectively. Here, we examine how information acquisition is modulated by social context, monetary cost, and the trustee’s trustworthiness. We gave participants the opportunity to sequentially sample information about a trustee’s reciprocation history before they decided whether or not to invest. Participants sampled less when there was a monetary cost and when the gathered information was more conclusive. On some trials, we induced a social context by telling the participant that the trustee would learn how much the participant sampled (“overt sampling”). Crucially, when sampling was free, participants sampled less when sampling was overt than when it was covert, suggesting that they avoided leaving negative impressions. We find that the data were well accounted for by a Bayesian heuristic model, in which the agent continues sampling until uncertainty about trustworthiness—as measured by the width of the posterior belief—drops below a level that they find tolerable. This study opens the door to broader applications of the tools and models of information sampling to social decision-making.

## Introduction

The processes of trust and reciprocity are essential to establish and maintain beneficial human cooperative interactions^1^. When trust is invested, and then reciprocated in turn, the investor (who makes the decision to trust) and the trustee (in whom trust is placed) typically emerge better off from the exchange. However, a trustee can often maximize their own profit by not reciprocating the trust placed in them. Therefore, a key factor in a successful cooperative interchange is the investor’s belief about whether or not the trustee will reciprocate. However, beliefs and the uncertainty that people have about their own beliefs are not directly observable. In addition, there are several strategies that people might use to update their beliefs when they gain information about a trustee. We therefore use computational models of information sampling to measure beliefs about trustworthiness and identify the cognitive mechanism by which those beliefs are updated. Using this approach, we study how this cognitive mechanism might be modulated when the trustee is aware of the information sampling.

Before elaborating on the effects of information sampling on the trustee’s willingness to reciprocate, we first define trust as a risky social decision under uncertainty. Some individuals are more trustworthy than others, and repeatedly interacting with the same person does not necessarily result in the same outcome^1,2^. In addition, we often only have limited information about someone’s level of trustworthiness. Therefore, decisions that involve trust are both risky and uncertain: there is variability in the outcome given a specific degree of trustworthiness of the other person (risk), and there is incomplete knowledge about this trustworthiness (uncertainty). Due to this uncertainty, it is beneficial to acquire information to determine whether someone can be trusted, which can be done by gathering information about the trustee’s past behaviour^3^.

However, there may be specific consequences to information sampling in a social context; your inquiries might affect the other person’s impression of you^4,5^. On the one hand, if someone continually checks up on our reliability, it may make us less likely to behave pro-socially with that person in the future, for example because too many detailed questions could be seen as offensive. As a consequence, the investor should gather *less* information when the trustee is aware of the inquiries than when they are not. On the other hand, extensive information search is typically indicative of deliberation or caution, which may be warranted if the decision is risky^6^. Therefore, the investor could alternatively attempt to communicate a strong aversion to betrayal by sampling *more* when the trustee is informed. This may be beneficial because reducing uncertainty about how a self-serving choice negatively impacts the well-being of another is shown to increase prosocial behavior, especially in those with high empathy^7^. Therefore, if the trustee realizes that the investor is highly betrayal-averse as indicated by extensive sampling prior to trusting, then the trustee may become more likely to reciprocate, especially if the trustee is highly empathic. Given this mechanism, the investor should gather *more* information when the trustee is aware of the inquiries. In view of these opposing predictions—sampling more to communicate betrayal aversion or sampling less to avoid leaving negative impressions—The goal of the study is to understand the cognitive mechanisms that underlie belief updates about the trustworthiness of other people. Specifically, we use computational models to distinguish between belief updates in normative and heuristic mechanisms, and we examine if these models can account for adjustments in information gathering in costly and social contexts.

## Methods

**Figure 1:**
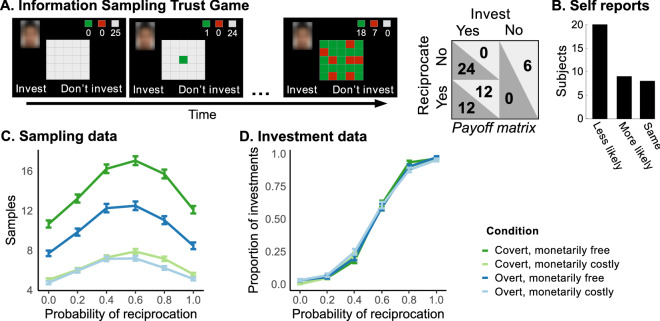
Task and data. (**A**) Trial sequence and payoff matrix in the Information Sampling Trust Game (ISTG). Before the participant made an investment decision, they could sequentially sample the decisions that the trustee made for other investors. On each trial, information could be sampled up to 25 times. The colour of the turned tile indicated the trustee’s past decision. Green = reciprocated trust, red = did not reciprocate trust, grey = not sampled. In the monetary cost conditions, 5 eurocents were deducted for each decision to sample (from a separate sampling budget of 125 eurocents per trial to keep the investment decision independent from sampling). In the monetarily free conditions, the 125 eurocents were always added to the trial outcome. Therefore, the monetarily costly condition did not have a higher gain. (**B**) Beliefs about the consequence of overt sampling. The most common belief amongst participants was that the more information they overtly sampled, the less likely reciprocation would become. Participants could choose from one of 3 categories: overtly sampling more information would make reciprocation less likely, more likely, or stay the same. (**C**) Sampling data which shows the effect of monetary cost and social context. Mean and SEM of drawn samples for each generative reciprocation probability per cost condition: green = covert sampling (trustee not informed), blue = overt sampling (trustee informed). (**D**) The proportion of investments increased with the probability of reciprocation for all conditions.

We designed a novel version of a single-shot trust game, the Information Sampling Trust Game (ISTG). Participants completed the ISTG in the investor role. On each trial participants (*n* = 37 (of which 12 men), age *m* = 22.95, *sd* = 3.71, range = 18–34) were endowed with €6 which they could either keep to themselves or invest in another player (the trustee). Not investing had no consequence and led to the next trial. Upon investing, the trustee received the endowment multiplied by 4 and would subsequently decide between either reciprocating (50–50 split) or defecting (keeping all €24). Crucially, before deciding whether or not to invest, participants were given the opportunity to sequentially gather information about a trustee’s previous reciprocation history by turning tiles in a 5 × 5 grid (Fig. 1). If a tile turned green the trustee had reciprocated money to a previous investor. If it turned red they defected to yet another investor. Deciding to either invest or not invest led to a new trial. Participants were told that there was a different trustee on each trial, the ratio red to green tiles on each trial could therefore vary, and that the location of the tile was not informative. Investment outcomes were not shown during the task. Unknown to participants, each trustee was computer-generated. The probability of a green tile was an independent draw from a Bernoulli distribution with parameter *r*, which was pseudo-randomly drawn from six values (0.0, 0.2, 0.4, 0.6, 0.8, and 1.0). The task consisted of 240 trials (10 per reciprocation probability, which were evenly distributed over the 4 conditions). This led up to a maximum of 240 × 25 tiles = 6,000 decisions.

The experiment consisted of four conditions that were varied within subjects: a monetary cost of sampling (either costly or cost-free), crossed with social context (overt or covert). In the overt conditions, we told the participant in the instruction phase that after the experiment, we would randomly select three trials. For the subset of these three trials on which the participant had decided to invest, we would contact the corresponding trustees and tell them how much the participant had sampled; then, the trustees would decide to either reciprocate or defect. We told the participant that their final pay-off would be the average of their earnings on the three selected trials. In reality, the trustees’ decisions were simulated using their respective reciprocation probabilities. All methods were carried out in accordance with the Declaration of Helsinki and approved by the local ethics committee (Commissie Mensgebonden Onderzoek regio Arnhem-Nijmegen 2014/288). Informed consent was collected from all participants.

### Descriptive statistics

The main focus of our study was on the computational models, which are cognitive process models that account for the data in a theoretically motivated manner. However, in this section we first use descriptive statistics to describe the general behavioural patterns in the data before we report the computational modelling results in the sections below. To this end, we used a mixed-effects model (see supplement Table S1 for full model) assessing the effects of monetary cost, social context, outcome uncertainty (variance of the Bernoulli distribution *r(*1-*r*)), and valence-dependency (*r*) on the number of samples. There was a significant interaction between monetary cost and social context (coefficients mean ± SEM; = 0.25 ± 0.02, *p* < 0.001); participants sampled less if sampling was overt and monetarily cost-free (= 0.33 ± 0.01, *p* < 0.001) and when sampling was overt and costly (= 0.07 ± 0.01, *p* < 0.001). As expected, people sampled more when the outcome uncertainty was larger (= 1.26 ± 0.05, *p* < 0.001), i.e., they sampled more when the acquired information was relatively inconclusive (as is the case when *r* is closer to 0.5, Fig. 1C). Outcome uncertainty interacted with the reciprocation probability *r* (= 0.55 ± 0.19, *p* = 0.003), which suggests that people sampled more when outcome uncertainty and reciprocation probability were high. We further explored whether the number of samples drawn depended on valence by  using Bonferroni corrected Wilcoxon signed-rank tests between symmetric pairs of generative probabilities. This revealed that people sampled more when *r* = 0.8 than when *r* = 0.2 (median difference = 0.775, *p* < 0.001) but not for *r* = 0.4 compared to *r* = 0.6 (median difference = 0.350, *p* = 0.11) or for *r* = 0 compared with *r* = 1 (median difference = 0.40, *p* = 0.013).

We then used a separate logistic regression to test whether the decision to invest was predicted by *r* and the conditions. This logistic regression returned a coefficient = 9.120.18 (*p* < 0.001) for *r*, indicating that the probability of investing increased with a higher *r*. This confirms that the acquired information was used in the investment decisions. Monetary cost, social context and their interaction were not significant predictors of the decision to invest (monetary cost: = -0.0230.095, *p* = 0.81; social context: = 0.0110.096, *p* = 0.91; interaction between monetary cost and social context: = 0.1130.135, *p* = 0.40).

After task completion, participants were asked to indicate whether they believed that when sampling was overt to the trustee, more sampling made reciprocation more likely, less likely, or would remain the same. Believing that overt information sampling would make reciprocation less likely was the most commonly reported answer (test for non-uniformity: χ^2^(2, *n* = 37) = 19.28, *p* < 0.001, Fig. 1D). Some subjects who responded in the less common category (that overt sampling would increase the probability of reciprocation) in fact also sampled less in overt compared to covert conditions (Fig. S1). Self-reports alone are therefore not sufficient to study the effects of overt sampling. Overall, the sampling data and the self-reports together suggest a social sampling cost of potentially leaving a negative impression on the trustee. This is consistent with the intuition that if someone continually checks up on our reliability, it may make us less likely to behave pro-socially with that person in the future.

### Computational models

**Figure 2:**
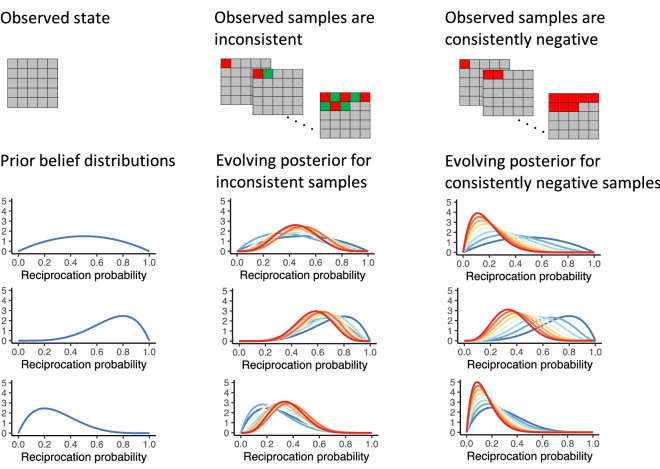
Illustration of belief updates depending on the prior belief distribution and the consistency of sample outcomes. The first column shows three possible prior distributions, which represent the agent’s beliefs in the absence of observations (nothing is sampled yet). The second column represents a scenario in which eight samples were alternately red and green. The three plots show the evolution of the posterior (from blue to red) based on the samples and the prior in the first column. Third column: the same as the second column but for eight consistently negative (red) samples.

To understand the cognitive mechanisms by which people sample information and the process by which overt sampling affects this mechanism we fitted normative and heuristic computational models. Each model describes different processes for how sampling is affected by the social and monetary cost conditions. We describe the intuition for these models and their differences here (see Supplement for formal descriptions). The first three models are Bayesian in the sense that the agent computes a posterior belief distribution over the trustee’s probability of reciprocation (Fig. 2).

We first consider a model which, analogous to the self-reports, assumes that every sample reduces the reciprocation probability by a constant factor. We refer to this model as the *Cost of Negative Impressions* (CNI) model. The model is normative in the sense that it maximizes expected utility. For every possible state (combination of red and green tiles), the agent uses the posterior belief to calculate the expected utility for every action: sampling, investing, and not investing. We derived the expected utilities of all state-action pairs using the Bellman equations and dynamic programming^8^. In the overt sampling conditions, the value of investing takes into account the factor ω (these are two free parameters: one for overt sampling under monetary cost and one for overt sampling without monetary cost) by which the agent believes the trustee’s reciprocation probability will decrease with each sample that is drawn. The model accounts for the immediate subjective cost of sampling, *c* (two free parameters: one for monetary cost condition and one for sampling without monetary cost as the latter may be non-zero due to the effort and time it takes to sample which can also be interpreted as a cost). We allow for two deviations from optimality as suggested by the (iterated) trust game literature: subjective prior beliefs^9^ and betrayal aversion^10^. The CNI model improved in fit to the data when these parameters were added (Table S2).

We refer to the second model as the *Sample cost* model. It is highly similar to the CNI model but more parsimonious in the sense that the agent simply thinks of all conditions as having an immediate subjective sampling cost, *c* that differs in weight for each of the four conditions. Similar to the CNI model we additionally tested the improvement in model fit when free parameters for a subjective prior belief and betrayal aversion were added (Supplement). While computing the value of information for each possible state in the task has a high precision, forward-reasoning models like the CNI model and the Sample cost model are likely too computationally expensive to be implemented as cognitive models. The brain might instead use a “good enough”, simpler heuristic strategy^11,12^. We therefore also examined the model fits of such computationally simpler strategies.

Our third model, the *Uncertainty model* reflects such a simpler, heuristic strategy. Similar to the CNI and Sample Cost models, the Uncertainty model uses a belief distribution over trustworthiness, which updates with each sample (Fig. 2). In the Uncertainty model, the agent continues sampling until uncertainty about trustworthiness—as measured by the standard deviation of the posterior belief distribution—drops below a level that they find tolerable. We refer to this uncertainty tolerance level as the criterion, *k*, which is a free parameter per condition. Note that uncertainty reduces faster when sample outcomes are consistent compared to when they are inconsistent (Fig. 2). We again tested the improvement in model fit when free parameters for a subjective prior belief were added. Here, the behavioral effect of betrayal aversion can be captured by the combination between the subjective prior and the criterion parameter *k*. Therefore, no additional betrayal aversion parameter was added.

Fourth, the *Threshold model* has an intuition similar to that of the standard Drift Diffusion Model^13^. Here, we consider the hypothesis that people do not use Bayesian posterior beliefs but instead maintain criteria for when they view a trustee’s behaviour as trustworthy or untrustworthy, and sample until the evidence meets one of those criteria. This requires keeping track of the sample outcomes in favour of investing and not investing. The decision to stop sampling information is then determined by whether their difference is sufficiently large, i.e., when the difference reaches a bound *b*. We allowed the bound to vary between the four conditions. It should be noted that the Threshold model is not equivalent to the DDMs that are often used in perception studies^13^ for the following reasons: First, in perception studies, the noise is typically Gaussian internal noise, whereas in our study, it is Bernoulli noise associated with past investment outcomes. Second, in perceptual applications, the time scale is hundreds of milliseconds to seconds, whereas here, accumulation takes place over a much longer time scale (tens of seconds). Finally, accumulation of evidence in regular DDMs is passive, whereas the Threshold model describes a process in which the agent decides at every time step. Based on the DDM literature, we tested versions with asymmetric bounds and collapsing bounds (Supplement).

### Computational modelling results

We compared the best fitting version of each model. For the CNI model and Sample cost model, these included a risk attitude term and a prior belief. For the Uncertainty model, we included a prior belief, and collapsing bounds for the Threshold model. The models were fitted to the data at the individual level using a log likelihood optimization algorithm as implemented in the fmincon routine in MATLAB (^©^Mathwork). The optimization was iterated 100 times with varying initiations to avoid local minima. Model recovery showed that the models were distinguishable (Table S3).

**Figure 3:**
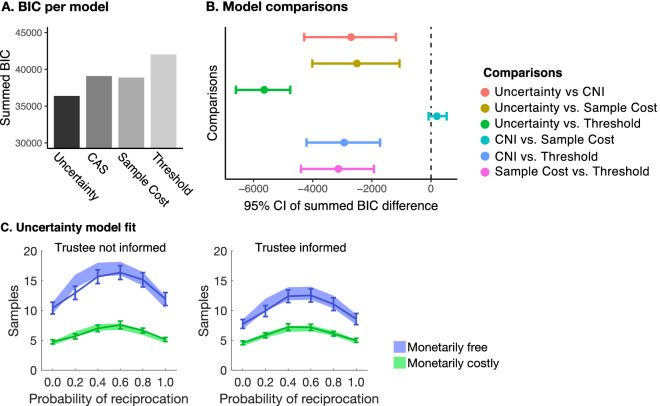
Model fit results. (**A**) The summed BIC for each model. (**B**) The 95%CI of the summed BIC difference between each model pair obtained using bootstrapping. Lower BIC values indicate a better fit. This shows that the Uncertainty model fitted best. (**C**) Uncertainty model fit. Line graph represents the raw data with SEM as error bars, shaded region is the model fit.

Overall, the Uncertainty model fitted best compared with the other models (Fig. 3; also see Supplement Table S2). The Threshold model fitted worse than all other models. Moreover, to test whether different individuals follow different models, we used Bayesian Model Selection^14,15^ (Table 1). This returned strong evidence in favor of the Uncertainty model as the most likely model in the population. It suggests that people use a heuristic rather than normative, forward-reasoning models for their decisions to sample. In addition, the better fit of the Uncertainty model over the heuristic Threshold model suggests that people use a posterior distribution over *r*, instead of using a non-Bayesian proxy in their sampling decisions.

**Table 1:** Bayesian Model Selection results.

Model	Expected frequencies	Exceedance probabilities
CNI	0.082	0.000
Sample Cost	0.310	0.029
Uncertainty	0.601	0.972
Threshold	0.007	0.000

Exceedance probabilities reflect the probability that a given model is more likely than any other model.

Next, we examined the parameter estimates of the winning Uncertainty model. These showed differences between conditions. Specifically, the criterion estimates confirmed that people were more tolerant to uncertainty when the trustee was informed of the sampling, and when sampling was monetarily costly (Wilcoxon signed-rank test all *p* < 0.005, see Table S3). The comparison between informed and not informed trustees reached significance when samples were monetarily cost free but not when they were monetarily costly. On average, people maintained prior means that were slightly negatively biased (median prior estimate = 0.475, Wilcoxon signed-rank returned: Z = -2.738, *p* = 0.006). This bias allowed the model to account for the empirical finding that people sampled more when the generative *r* was larger than 0.5 compared to when it was smaller than 0.5.

In sum, model comparisons demonstrated that people sample until uncertainty drops below a subjective uncertainty criterion. This subjective criterion depended on the posterior belief distribution over reciprocation probabilities. Interestingly, the Threshold model fitted least well, even when using collapsing or asymmetric bounds. This supports our interpretation that people use a posterior distribution for their sampling decisions, because the Threshold models represent simple heuristics, similar to the Uncertainty model but without a posterior belief distribution.

We further examined whether the winning Uncertainty model could also predict trust decisions (after sampling has concluded) by using the parameter estimates. We fitted the expected utilities that resulted from the Uncertainty model to the trust decisions, allowing for bias and decision noise temperature (Eq. 9). This showed that the Uncertainty model significantly predicted of the probability of trusting (Uncertainty model *p* < 0.001, Nagelkerke pseudo-R^2^ = 0.882). However, so did all other models (CNI model *p* < 0.001, Nagelkerke pseudo-R^2^ = 0.643; Sample Cost model *p* < 0.001, pseudo-R^2^ = 0.832; Threshold model, *p* < 0.001, Nagelkerke pseudo-R^2^ = 0.733), suggesting that all models could predict trust decisions after sampling had concluded.

### Study 2

**Figure 4:**
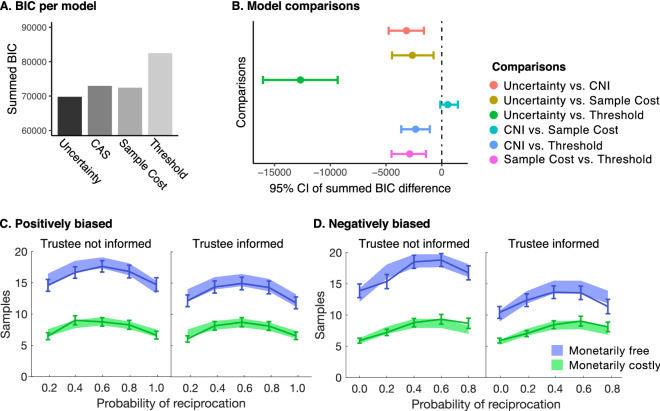
Model fit results for study 2. (**A**) The summed BIC for each model. (**B**) The 95%CI of the summed BIC difference between each model pair obtained using bootstrapping. Lower BIC values indicate a better fit. This shows that the Uncertainty model fitted best. (**C**) The Uncertainty model fit for the positively biased data. The line graphs represent the raw data and SEM, which show that the effect of condition replicates in both biased conditions. Shaded region is the model fit. (**D**) The Uncertainty model fit for the negatively biased data.

To test the robustness of our findings under variations of the distribution of the reciprocation probability, we conducted a second, independent study (*n* = 75) with biased generative distributions of *r*. The experimental procedure was identical to study 1, apart from the fact that participants sampled over either positively biased (*r* = 0.2, 0.4, 0.6, 0.8, and 1.0), or negatively biased (*r* = 0.0, 0.2, 0.4, 0.6, and 0.8) generative probabilities of reciprocation. We examined the number of samples as a function of bias, monetary cost, social context, outcome uncertainty, and reciprocation probability in a mixed-effects model (see Supplement for the model specification and full results table). We replicated all effects in study 1. However, here we found that the effect of social context was present when sampling was free (= 0.2000.016, *p* < 0.001), but was not significant when it was costly (= 0.0200.016, *p* = 0.225; Fig. 4C,D). The subjective reports in study 2 also replicated the pattern in study 1 (Fig. 4, Table S6). For the computational models, within-model comparisons replicated for all models except for the Threshold model, where asymmetric bounds improved the model fit (Supplement). The between-group variational Bayesian analysis showed that the winning model did not differ between the positive and negatively biased groups (probability that the two groups have the same model frequencies = 0.930). Between model comparisons suggested that the Uncertainty model performed best (Figure A and B also see Table S7) and the uncertainty tolerance parameter estimates varied as a function of condition further replicating the results of Study 1 (Table S8).

We found the following expected frequencies of the models in the population: 0.15 for the CNI model, 0.233 for the Sample Cost model, 0.533 for the Uncertainty model and 0.08 for the Threshold model. The exceedance probabilities returned 0.00 for the CNI model, 0.00 for the Sample Cost model, 0.99 for the Uncertainty model and 0.00 for the Threshold model. This again shows that the Uncertainty model is more likely than the other two models. The fitted models predicted the trust decisions after sampling had concluded: Uncertainty model (*p* < 0.001, Nagelkerke pseudo-R^2^ = 0.64); the CNI model (*p* < 0.001, Nagelkerke pseudo-R^2^ = 0.40); Sample Cost model (*p* < 0.001, Nagelkerke pseudo-R^2^ = 0.15); the Threshold model (*p* < 0.001, Nagelkerke pseudo-R^2^ = 0.69).

## Discussion

Acquiring information to reduce our uncertainty about the intentions and future actions of others is crucial for our social behaviour. Understanding why and about whom we gather information has been the objective of many studies in social psychology^16^. However, one often ignored aspect of social information sampling is that people might consider the fact that the other is aware of the inquiries and adjust their sampling accordingly. Here, we used computational modelling to examine the cognitive mechanisms of trustworthiness information sampling and how overt sampling changes this process. Across two studies, we showed that people used a heuristic cognitive strategy for sampling. Specifically, we demonstrated that people sample until their uncertainty drops below an uncertainty tolerance criterion. Moreover, we found that people became more uncertainty tolerant when the quantity of acquired information was overt to the trustee. The self-reports were consistent with this result, people believed that sampling would decrease the reciprocation probability.

We believe that our overt sampling finding has interesting implications. It shows that when people gather information about another person, they factor in that these inquiries may change that person’s behavior towards them. This implies that learning about others diverges in important ways from sampling information about the statistics of a non-social environment. Specifically, seeking information about others requires thinking about the effects that one’s inquiries may have. Reasoning about how other people’s behavior may change as a function of your own actions therefore relies on theory of mind. This is further supported by participants’ self-reports, which indicated that they believed that information sampling would decrease the trustee’s probability of reciprocation. Our computational modelling results show that this belief about social context is not implemented in a normative manner. Instead people became more uncertainty tolerant, which suggests a heuristic, computationally cheaper strategy. However, a small subset of subjects also reported beliefs that contradicted their behavior. Self-reports of strategy and performance are challenging and depend on metacognitive abilities^17^. This finding shows that self-reports alone are not sufficient for studies that aim to expose the cognitive mechanisms that govern human behavior.

An interesting question in this regard is whether believing that overt sampling reduces the trustee’s reciprocation probability is in fact adaptive, even though it counterintuitively leads to decisions being made using less information. Previous work might shed some light on this question. Looking for better options while in a potentially reciprocal partnership will eventually decrease the probability of the continuation of that partnership^4,18,19^. People then often decide not to overtly look for better options in order to signal to their partner that their cooperative behavior is unconditional, i.e. independent of the value of the “temptation” to defect^4^. Our findings extend this work in important directions, as in our task information was sampled about the trustee’s decision history itself, instead of about other potentially tempting options. We showed that people reduce their quantity of information sampling to avoid leaving negative impressions. This social information sampling cost thereby demonstrates that the appearance of being trusting contributes to the decisions to acquire information for the purpose of building trust.

A potentially detrimental consequence of sampling less to avoid leaving negative impressions is that the subsequent investment decisions are less well informed. In particular, prior beliefs about someone’s trustworthiness based on facial judgement of trustworthiness, attractiveness, narratives about moral character, and previous social experiences in unrelated settings, can bias learning about trustworthiness^9,20-22^. If an initial sample is consistent with the prior information (such as when a sample is consistent with prior trustworthiness judgements based on attractiveness), then it is more likely that the investor will stop sampling after that sample. This can be a missed opportunity if the initial sample is not representative of the actual trustworthiness (e.g., when the trustee is actually more trustworthy). Given that avoiding negative impressions reduces information search, the subsequent decision to trust or not might become even more susceptible to biased prior beliefs. Future studies are required to test if avoidance of negative impressions will indeed make people more sensitive to prior information, especially prior information that has very little or highly uncertain diagnostic value, such as facial attractiveness.

Independent of the overt sampling effect, participants were clearly sensitive to outcome consistency. People gathered most information when the absolute difference between green and red tiles was small. We also observed a subtle valence-dependent asymmetry in sampling. Specifically, participants sampled more when outcomes were variable and mostly positive, than when outcomes were variable and mostly negative. Others have shown that such a valence-dependency can create a bias in belief updates, because people stop sooner when the initial sample outcomes are negative, thereby not discovering that these first samples were in fact not representative of the actually more positive reciprocation probability^23^. This valence-dependency is possibly consistent with well-established findings in impression formation and person perception, in which negative behavior is generally viewed as more diagnostic of morality than positive behavior^24-27^. Those studies show that negative evidence is especially more heavily weighed when behavior is extreme^25^. Future studies are needed to investigate whether a negativity bias in trustworthiness information search interacts with information consistency when the moral transgressions are relatively minor, such that people are especially uncertainty intolerant when others are mostly trustworthy but not consistently so.

We told subjects that the trustee would be informed of the sampling. In many daily-life scenarios (e.g. gossip), people are uncertain that the trustee will learn about the inquiries. Future studies could inform the trustee about sampling behaviour in a stochastic manner. The model is straightforwardly extended to this situation: the utility of investing will become a linear combination of two terms. Another potential extension could involve directly probing trustee behaviour. Understanding whether and how trustees change their behaviour when investors gather information is important to understand whether avoiding to leave negative impressions by sampling less is truly adaptive. Moreover, future studies are needed to identify the nature of such negative impressions. For example, overt sampling might signal suspicion of the trustee. In addition, trustees might view overt sampling as a signal that the trustee is motivated by monetary gain instead of the prosocial act of sharing. These interpretations could of course go hand in hand, as people who are more motivated by extrinsic reward may also be more suspicious of others. Both of these interpretations could reduce the trustee’s willingness to reciprocate. Further study of the trustee’s behaviour will provide more detailed insight into their potentially negative impressions.

More broadly, our study further illustrates the value of computational modelling in social decision-making^28^. Descriptive statistical models primarily map input to output and do not have the ability to give insights into the underlying cognitive mechanisms. The computational modelling results show that people use belief distributions over the trustworthiness of others. We rejected the threshold model which did not rely on belief distributions. Our other computational models distinguished between alternative mechanisms in how this belief distribution is used to make information sampling decisions. We found that the decision rule that people use to stop sampling is uncertainty based. Our models managed to capture people’s motivation to avoid leaving a negative impression on trustees when sampling. This leads to new opportunities to study the trustee’s viewpoint, such as how the trustee’s impression of the investor changes when observing the investor’s sampling behaviour, and how that impression subsequently changes the trustee’s willingness to reciprocate.

Our findings may provide a benchmark to uncover social reasoning aberrations in psychiatric disorders. Some psychiatric disorders can be characterized by biases in information sampling, such as insufficient information gathering in addiction^29^, asymmetric weighting of negative evidence in depression^30^, and impaired information sampling cost signals associated with compulsivity^31,32^. Importantly, specific impairments in the ability to consider the mental state of others or to respond to social signals are central to a range of psychiatric disorders, including borderline personality disorder and autism spectrum disorder^33-36^. In a separate study, we used an abbreviated version of the current task and revealed developmental changes in belief updates during adolescence^37^. This can provide a particularly relevant benchmark as the onset of many psychiatric disorders takes place during adolescence^38^. More generally speaking, our study opens the door to broader applications of the tools and models from information sampling to understand social decision-making.

## Supplementary information

Supplementary information
