# Supplementary material for: The social cost of gathering information for trust decisions
Source: Sci Rep. 2020 Aug 21;10:14073. doi: 10.1038/s41598-020-69766-6 (PMC7442811; doi:10.1038/s41598-020-69766-6)
Supplement: Supplementary file 1 — Supplementary information [file 41598_2020_69766_MOESM1_ESM.docx]

# The Social Cost of Gathering Information for Trust Decisions

*Ma, I.^1,3^, Sanfey, A. G.^1,2^, Ma, W.J.^3^

^1^Donders Institute, Radboud University, The Netherlands

^2^Behavioural Science Institute, Radboud University, Nijmegen, The Netherlands

^2^New York University, New York, USA

**ONLINE SUPPLEMENTARY INFORMATION**

*IST trial procedure*

At the start of each trial, participants started with an investment endowment of 6 euros (E6) and a sampling budget of 25 x 5 eurocent. A trial started with the presentation of a starting screen that contained:

- A blurry photo of a person (the trustee). A black bar partially covered the trustee’s photo in the social context in which the trustee would be informed of the sampling (overt sampling).
- A visualization of the condition: in the no cost condition, the word “free” was displayed, and in the cost condition, an image of tokens and a budget counter at 25.
- A 5-by-5 grid of covered tiles.
- A visualization and numerical representation of the initial counts of positive samples (green tiles; 0), negative samples (red tiles; 0), and covered tiles (grey; 25).

The participant generally had three options: to click on a tile to turn it, to choose “Invest E6”, or to choose “Do not invest”. If the number of unturned tiles was 0, then only the latter two options were available. There was no time limit for the decision. If the participant clicked on an empty square, its color was revealed. The color of the turned tile was determined by a draw from a Bernoulli distribution with parameter equal to the reciprocation probability *r* on that trial, *r* (0, 0.2, 0.4, 0.6, 0.8, or 1). Simultaneously with the revealing of the tile’s color, the three counters were correspondingly updated, and in the monetary cost condition, 5 eurocents were subtracted from the sampling budget counter. The participant then made their next choice, still within the same trial. Once the participant clicked “Invest E6” or “Do not invest”, a confirmation of their choice was shown for 1.5 seconds on an otherwise blank screen, and the next trial began. Outcomes of the investment decisions were not shown during the task.

*Descriptive statistics and mixed model*

Participants sampled on average of 11.5±0.5 times per trial (from a maximum of 25). Descriptive statistics were obtained using a mixed-effects model in R, package lme4 ([www.r-project.org](http://www.r-project.org)^1,2^ R Core Team, 2014). We used a mixed-effects model (applying the lmer() function in R) to test whether the number of samples differed as a function of monetary cost, social context, outcome uncertainty, and reciprocation probability. For the dependent variable we log transformed the number of samples per trial, per participant (first adding 1 to the number of samples to avoid -infinity in the rare case that a participant had not sampled on a trial). We dummy coded both the monetary cost and social context condition levels (0 and 1). Outcome uncertainty was operationalized as the variance in the Bernoulli distribution given by *r*(1-*r*), where *r* is the reciprocation probability that generates the outcomes in the grid. The mixed-effects model included the interaction between monetary cost and social context, the interaction between outcome uncertainty and reciprocation probability, and a random intercept per participant.

TableS1. Results mixed-effects model study 1

|  | **Log(samples)** | | | |
| --- | --- | --- | --- | --- |
| *Predictors* | *Estimates* | *95%CI* | *Statistic* | *p* |
| (Intercept) | 2.30 | 2.18 – 2.42 | 36.70 | **<0.001** |
| MonetaryCost | -0.62 | -0.65 – -0.60 | -45.19 | **<0.001** |
| SocialContext | -0.33 | -0.36 – -0.31 | -24.27 | **<0.001** |
| Uncertainty | 0.98 | 0.78 – 1.19 | 9.40 | **<0.001** |
| ReciprocationProb | 0.09 | 0.05 – 0.12 | 5.09 | **<0.001** |
| MonetaryCost * SocialContext | 0.26 | 0.22 – 0.30 | 13.19 | **<0.001** |
| Uncertainty * ReciprocationProb | 0.55 | 0.19 – 0.91 | 2.96 | **0.003** |
| **Random Effects** | | | | |
| σ^2^ | 0.22 | | | |
| τ_00_ _ppnr_ | 0.15 | | | |
| ICC | 0.40 | | | |
| N _ppnr_ | 40 | | | |
| Observations | 9440 | | | |
| Marginal R^2^ / Conditional R^2^ | 0.200 / 0.519 | | | |

Output produced using the tab_model function in R

*Self-reports*

In terms of the self-reports, participants indicated whether they believed that overt sampling would make the trustee’s reciprocation: a) less likely, b) stay the same, c) more likely. However, the self-reported response category was not predictive of the difference in number of samples between covert and overt conditions (Kruskal-Wallis *H* = 4.61, *p* = 0.100). This might be because self-reports are noisy, in part because they rely on meta-cognitive abilities. We therefore use the frequencies of the self-reports as inspiration for one of our models and will not analyze the self-reports any further.

Figure S1. The y-axis shows the main effect of social context. Each transparent dot represents one subject. For each subject, we calculated the difference between the total number of samples in the covert condition and the overt condition and divided this by the number of trials to obtain the mean difference. Mean differences higher than 0 indicate a stronger main effect (i.e., more samples in the covert than in the overt condition). The boxplot shows that not all subjects who responded that sampling would make the trustee more likely to reciprocate actually sampled more. The median was not below 0 in the “more likely” category.

**Computational models**

*General structure of models*

The state is determined by the number of turned green tiles, *n*_+_, and the number of turned red tiles *n*_-_. We consider four models, all with their own variants: A Cost of Negative Impressions (CNI) model, a Sample cost model, an Uncertainty model, and a Threshold model. In each model, the agent computes a decision variable based on *n*_+_ and *n*_-_, and the probability to stop sampling is a noisy function of this decision variable. After the agent stops sampling, the same decision variable determines the probability that the agent invests. The CNI model, the Sample cost model, and the Uncertainty model are based on an evolving posterior distribution over the trustee’s reciprocation probability *r*.

*Cost of Negative Impressions (CNI) model*

The CNI model is based on the agent computing expected utility through forward reasoning. It consists of four components: prior beliefs over reciprocation probability *r*, an evolving posterior distribution over *r*, iterative maximization of future expected utility under this distribution, and decision noise. We assume that the prior over *r* is a beta distribution with parameters *α*_0_ and *β*_0_. The posterior over *r* is a beta distribution with parameters $\alpha=n_{+}+\alpha_{0}$ and $\beta=n_{-}+\beta_{0}$.

An investment outcome can have two values: reciprocation (outcome = 1, then the investment amount is multiplied by *m* = 2) or betrayal (outcome = 0, then the investment amount is multiplied by *m* = 0). The investment outcome follows a Bernoulli distribution with parameter *r*, i.e *p*(outcome=1|*r*) = *r*. When building a belief about the outcome of an investment decision, the agent does not know *r* and therefore has to marginalize over *r* using the current posterior over *r*, which is *p*(*r*|*α*, *β*). This gives the conditional distribution of outcome given *α* and *β*:

$p\left( \mathrm{outcome}=1 \right|\alpha,\beta)= \int p\left( \mathrm{outcome}=1 | r \right)p\left( r | \alpha,\beta\right)dr=\int rp\left( r | \alpha,\beta\right)dr=\frac{\alpha}{\alpha+\beta}$ (1)

and:

$p\left( \mathrm{outcome}=0| \alpha,\beta\right) = \frac{\beta}{\alpha+\beta}$.

This is a distribution with mean $\frac{\alpha m}{\alpha+\beta}$ and variance $\frac{\alpha\beta m^{2}}{\left( \alpha+\beta\right)^{2}}$.

We hypothesize that the agent believes that overtly sampling induces a decrease in the reciprocation probability *r*. Since people sample over the trustee’s *past* decisions, such a belief does not affect the probability of a green *sample* outcome. However, if the investor invests after terminating their sampling, the reciprocation probability that the investor believes they will experience is not the *r* from the trustee’s past (the one that the investor has worked to infer through sampling), but a modulated *r*, where every sample that the investor has drawn multiplies *r* by a factor ω that takes values between 0 and 1. Thus, if the investor has drawn *n* samples, the reciprocation probability is:

$$\begin{aligned} r'= \omega^{n}r\#\left( 2 \right) \end{aligned}$$

The posterior distribution over *r*’ is now a rescaled and truncated beta distribution. The distribution *p*(outcome|$\alpha,\beta$) now has a mean of $\frac{m\alpha\omega^{n}}{\alpha+\beta}$ and a variance of $\frac{m^{2}\alpha\beta\omega^{n}}{\left( \alpha+\beta\right)^{2}}$. The expected utility of investing gets modified accordingly: We fit one $\omega$ for each of the two overt condition (when the trustee is informed of the sampling), that is, once for overt sampling without a monetary cost, and once overt sampling with a monetary cost. The weight parameter $\omega$ is 1 when sampling is covert sampling (i.e., when the trustee is not aware of the sampling).

We are now ready to define the expected utility of not investing and investing. The expected utility of not trusting is *U*_0_ = 1, the normalized endowment, which is independent of *α* and *β.* The expected utility of trusting should contain the expected amount earned from an investment. In addition, people are also known to differ in their risk attitude. Specifically in trust games, people may be betrayal-averse ^37^. To model such a risk attitude, we subtract a multiplier times the variance of the amount earned from the expected amount earned. The overall expected utility of trusting, which we denote by *U*_1_, is weighted by the decrease in *r*, and thus becomes:

$$\begin{aligned} U_{1}\left( \alpha,\beta\right) =\omega E\left[ \mathrm{outcome} | \alpha,\beta\right]- \omega^{2}\lambda\mathrm{Var}\left[ \mathrm{outcome} | \alpha,\beta\right]\#\left( 3 \right) \end{aligned}$$

$$= \frac{m\omega\alpha}{\alpha+\beta} - \frac{\lambda m^{2}\omega^{2}\alpha\beta}{\left( \alpha+\beta\right)^{2}}$$

where *λ* parametrizes risk attitude, with *λ*>0 representing risk aversion and *λ*<0 risk-seeking.

At any time, the participant has two possible actions: stopping (*a* = 0) and sampling (*a* = 1), except when *t* = *T*+1, when only *a* = 0 is available. The expected value of a state-action pair, *Q*($\alpha$, *β*; action), is given by the Bellman equations^38^. Specifically, if at time *t*, the participant stops (*a* = 0), then the expected value of the state (*α*, *β*) is the higher of the expected utilities of not investing and investing:

$$\begin{aligned} Q_{t}\left( \alpha,\beta;a=0 \right)=\max\left\{ U_{0},U_{1}\left( \alpha,\beta\right) \right\}\#\left( 4 \right) \end{aligned}$$

When *t* = *T*+1, the value of the state (*α, β*) is $V_{T+1}\left( \alpha,\beta\right)=Q_{T+1}(\alpha,\beta;0)$. At earlier times, *V_t_* is the larger of the two expected utilities:

$$\begin{aligned} V_{t}\left( \alpha,\beta\right)=\max\left\{ Q_{t}\left( \alpha,\beta;a=0 \right),Q_{t}\left( \alpha,\beta;a=1 \right) \right\}\#\left( 5 \right) \end{aligned}$$

The expected value of a sampling action at time *t* in the state *α,* *β* by $Q_{t}(\alpha,\beta;a=1)$ is:

$$\begin{aligned} Q_{t}\left( \alpha, \beta; a=1 \right)=\frac{\alpha V_{t+1}\left( \alpha+1,\beta\right)+\beta V_{t+1}\left( \alpha, \beta+1 \right)}{\alpha+ \beta}-c\#\left( 6 \right) \end{aligned}$$

where *c* is the subjective cost of a sample. We fit one cost *c* for each monetary cost condition, i.e., once for when it is monetarily cost free to sample, and once when sampling is monetarily costly (irrespective of overt or covert sampling).

By starting with the final state (when *n* = *T*) we can apply the equation for the expected utility of stopping (equation 4 for *t* = *T*+1, $Q_{t}(\alpha,\beta;a=0)$). We can then work our way back in time, to obtain the optimal solution for every possible state (dynamic programming^3^). The decision variable, denoted by DV, is the difference between the utilities of sampling and stopping:

$$\begin{aligned} DV\left( \alpha,\beta\right)=Q_{t}\left( \alpha,\beta;a=1 \right)-Q_{t}\left( \alpha,\beta;a=0 \right)\#\left( 7 \right) \end{aligned}$$

The optimal policy would be to sample when the DV is positive; however, we will also introduce decision noise temperature.

*Decision noise*

The model allows for decision noise through a logistic function:

$$\begin{aligned} p\left( sample | \alpha,\beta\right)=\frac{1}{1+e^{-\frac{DV\left( \alpha,\beta\right)-k}{\tau}}}\#\left( 8 \right) \end{aligned}$$

where *DV* is the decision variable in the model, *k* is a criterion parameter, andτ is the decision noise (higher *τ* means more noise). The noise is not part of the forward reasoning calculations. Instead, it is part of the decision to sample or stop once the optimal solution is derived.

*Investment decisions with decision noise*

For the models that use a Bayesian belief distribution (the CNI model, the Sample cost model and the Uncertainty model described below), the equation for the utility of investing is the same. Once the decision to stop sampling has been made, the agent choses to invest using the utility of investing with decision noise:

$$\begin{aligned} p\left( \mathrm{invest} | \alpha, \beta\right)=\frac{1}{1+e^{-\frac{U_{1}\left( \alpha,\beta\right)-k}{\tau}}} \end{aligned}(9)$$

Note that we allow for a different temperature in the probability of sampling in all models.

*Sample cost model*

This model is similar to the CNI model without weight parameter $\omega.$ The Sample cost model is based on the agent computing the expected utility via forward reasoning and assumes that there is a cost for each sample, which varies between the four conditions. Thus, instead of a decrease in the reciprocation probability *r*, there is a separately estimated cost parameter c for each of the four conditions (equation 6). Formally, the model is identical to the CNI model with exception of the utility of investing, which becomes:

$$\begin{aligned} U_{1}\left( \alpha,\beta\right) =E\left[ \mathrm{outcome} | \alpha,\beta\right]- \lambda\mathrm{Var}\left[ \mathrm{outcome} | \alpha,\beta\right] \\ = \frac{m\alpha}{\alpha+\beta} - \frac{\lambda m^{2}\alpha\beta}{\left( \alpha+\beta\right)^{2}} \\ \#\left( 10 \right) \end{aligned}$$

The resulting policy is ran through a softmax to account for decision noise (equation 9).

*Uncertainty model*

The Uncertainty model is based on a criterion on the uncertainty about *r*. Similar to the CNI model, the prior over *r* is a beta distribution with parameters *α*_0_ and *β*_0_ and the posterior over *r* is a beta distribution with parameters $\alpha=n_{+}+\alpha_{0}$ and $\beta=n_{-}+\beta_{0}$. The decision variable in the Uncertainty model is the standard deviation of this posterior distribution:

$$\begin{aligned} DV_{Uncertainty}\left( \alpha,\beta\right)=\sqrt{\frac{\alpha\beta}{\left( \alpha,\beta\right)^{2}\left( \alpha+\beta+1 \right)}}\#\left( 11 \right) \end{aligned}$$

Decision noise is again modelled using equation (9).

*Threshold model*

In the Threshold model, the agent keeps track of the absolute difference between green and red tiles and stops sampling when this difference reaches a bound. However, to be consistent with our other models and with most of the value-based decision literature, we will use soft rather than hard bounds:

$$\begin{aligned} DV_{\mathrm{Threshold}}\left( n_{+},n_{-} \right)=\left| n_{+}-n_{-} \right|\#(12) \end{aligned}$$

The probability that the agent stops sampling is a logistic function of the difference between this decision variable and a bound *b*:

$$\begin{aligned} p\left( \mathrm{sample} | n_{+}, n_{-} \right)=\frac{1}{1+e^{-\frac{DV\left( n_{+}, n_{-} \right)-b}{\tau}}}\#\left( 13 \right) \end{aligned}$$

The parameter τ is the temperature of the decision noise. If *τ* approaches 0, the mapping from *DV*_Threshold_ to the decision becomes deterministic: stop sampling when |*n*+-*n*-| > *b*. The presence of decision noise means that the agent sometimes continues sampling even when |*n*_+_-*n*_-_| > *b*, and sometimes already stops sampling when this inequality is not met.

We consider three versions of the Threshold model, which differ in the assumptions about the bound *b*. In the Threshold_s_ model (where “s” stands for “symmetric”), *b* is a fixed constant. In the Threshold_a_ model (where “a” stands for “asymmetric”), *b* takes three possible values, depending on the sign of *n*_+_-*n*_-_:

$$\begin{aligned} b=\left\{ \begin{matrix} b_{+} & \mathrm{if}n_{+}>n_{-} \\ \frac{b_{+}+b_{-}}{2} & \mathrm{if}n_{+}=n_{-} \\ b_{-} & \mathrm{if}n_{+}< n_{-} \end{matrix} \right.\#\left( 14 \right) \end{aligned}$$

One consequence of this model is that when the difference between green and red tiles is close to zero, this model will take a very long time to decide between investing or not investing. To prevent that the agent samples for too long, it may be optimal that the bounds of investing and not investing “collapse” towards zero over time^4^. We implemented the collapsing bounds, in the Threshold_c_ model (where “c” stands for “collapsing”), by exponentially decaying the bounds towards zero with speed *s* over time, where time is defined as the number of samples *n*:

$$\begin{aligned} b_{c}=b* e^{-s*n}\#\left( 15 \right) \end{aligned}$$

where *s* is a free parameter. If *s* is zero, this term is equivalent to a non-collapsing bound.

*Investment decisions threshold model*

Once the decision to stop sampling has been made, the agent choses to invest using the utility of investing with decision noise:

$$\begin{aligned} p\left( \mathrm{invest} | \alpha, \beta\right)=\frac{1}{1+e^{-\frac{n_{+}- n_{-}-k_{\mathrm{invest}}}{\tau_{\mathrm{invest}}}}}\#\left( 16 \right) \end{aligned}$$

Note that we allow for a different temperature in the probability of sampling in all models.

**Within-model comparisons**

We first compared different versions of each model by adding theoretically motivated free parameters and assessing the improvement in model fit in terms of 95%CI in the AIC and BIC. We then compared the best version of each model for the between-model comparisons.

The basic *CNI model* has six free parameters; decision noise temperature, decision bias, one sample cost parameter for each of the two levels of monetary cost (costly and free), and one weight parameter for the cost of leaving negative impressions for the two conditions in which the trustee is informed (informed, costly; informed, free). In our paradigm, an individual’s initial belief about trustworthiness is a prior probability distribution over the probability of a reciprocation (*r*), before any information about the trustee is known. We modelled the prior as a beta distribution with two free parameters (*α*_0_ and *β*_0_). We operationalized risk attitude by subtracting the variance of the outcome, multiplied by a free parameter *λ*, from the expected amount earned from investing to obtain the utility of the decision to invest. The introduction of the subjective prior yielded a large improvement over the basic model (Table S2, within model comparisons). The risk attitude parameter yielded further improvement (Table S2, within model comparisons).

The basic *Sample cost model* has six free parameters; decision noise temperature, decision bias, and one parameter for the cost of a sample in monetary units in each of the four experimental conditions. In our paradigm, an individual’s initial belief about trustworthiness is a prior probability distribution over the probability of a reciprocation (*r*), before any information about the trustee is known. We modelled the prior as a beta distribution with two free parameters (*α*_0_ and *β*_0_). We operationalized risk attitude by subtracting the variance of the outcome, multiplied by a free parameter *λ*, from the expected amount earned from investing to obtain the utility of the decision to invest. The introduction of the subjective prior yielded a large improvement over the basic model (Table S2, within model comparisons). The risk attitude parameter yielded further improvement (Table S2, within model comparisons).

In the *Uncertainty model*, the agent computes the posterior in the same way as the CNI and Sample cost model, but the subject samples until uncertainty – measured as the posterior standard deviation – drops below a fixed criterion. The basic Uncertainty model has five free parameters: one decision noise parameter and one criterion parameter for each of the four experimental conditions. For the same reasons as described earlier, we tested the improvement in model fit when adding free priors. We found that allowing for a subjective prior improved the model fit.

The basic version of the *Threshold model* has five free parameters; decision noise temperature, and one bound for each of the four experimental conditions. Next, we sequentially examined two versions of the Threshold model. First, we allowed for potential valence-dependent biases in information sampling^5–7^ by examining the model fit improvement of the Threshold model with asymmetric bounds for green and red tiles. Second, we tested the model when the bounds collapse to zero over time. This is a common approach to avoid excessive sampling when there is high stochasticity in outcomes, and may be optimal^4,8^. The speed of this collapse is determined by a free parameter *s*. We find that neither collapsing bounds nor asymmetric bounds significantly improved model fit. However, we used the version with collapsing bounds (Threshold_c_) for the between-model comparisons in Study 1, as this was the Threshold model version with the best loglikelihood and therefore the most stringent test for the between model-comparisons.

Table S2. Model comparisons study 1

|  | | | **AIC** | | | | **BIC** | | |
| --- | --- | --- | --- | --- | --- | --- | --- | --- | --- |
|  |  | | | 95% CI | |  | | 95% CI | |
|  | | sum | | lower | upper | sum | | lower | upper |
| **Within-model comparisons** | |  | |  |  |  | |  |  |
| CNI basic vs. priors | | 1779 | | 1322 | 2261 | 1366 | | 911 | 1848 |
| CNI priors vs. risk attitude | | 969 | | 420 | 1620 | 762 | | 216 | 1415 |
| Sample cost basic vs. priors | | 2006 | | 1506 | 2550 | 1584 | | 1085 | 2128 |
| Sample cost priors vs. risk attitude | | 1062 | | 615 | 1569 | 851 | | 404 | 1360 |
| Uncertainty basic vs. priors | | 1923 | | 1211 | 2719 | 1501 | | 786 | 2300 |
| Threshold_s_ vs. Threshold_a_ | | 1005 | | 463 | 1651 | 161 | | -380 | 805 |
| Threshold_a_ vs. Threshold_c_ | | 546 | | -63 | 1240 | -87 | | -692 | 604 |
| **Between-model comparisons** | |  | |  |  |  | |  |  |
| CNI vs. Sample cost | | 199 | | -81 | 528 | 199 | | -81 | 528 |
| CNI vs. Uncertainty | | 2292 | | 777 | 3877 | 2705 | | 1188 | 4292 |
| CNI vs. Threshold | | -3762 | | -5029 | -2554 | -2935 | | -4210 | -1719 |
| Sample cost vs. Uncertainty | | 2093 | | 655 | 3596 | 2506 | | 1065 | 4013 |
| Sample cost vs. Threshold | | -3961 | | -5217 | -2765 | -3134 | | -4397 | -1930 |
| Uncertainty vs. Threshold | | -6054 | | -7010 | -5171 | -5640 | | -6597 | -4759 |

95% CI = Bootstrapped 95% confidence interval of the summed difference between model fits. Smaller AIC and BIC values indicate better fit. Thus, positive values indicate a better fit for the second model. The models were fitted to the data at the individual level using a log likelihood optimization algorithm as implemented in the fmincon routine in MATLAB (©Mathworks). The optimization was iterated 100 times with varying initiations to avoid local minima. Because of the summation of the difference, large positive or negative numbers therefore reflect that one model wins consistently, i.e. for most subjects.

Table S3. Between-condition estimates for the heuristic models study 1

|  | | Trustee not informed | | | Trustee informed | | |
| --- | --- | --- | --- | --- | --- | --- | --- |
|  |  | | 95% CI | |  | 95% CI | |
|  | | median | lower | upper | median | lower | upper |
| Uncertainty model *k* monetarily free | | 0.028 | 0.022 | 0.053 | 0.075 | 0.041 | 0.092 |
| Uncertainty model *k* monetarily costly | | 0.119 | 0.105 | 0.131 | 0.122 | 0.112 | 0.129 |

Bootstrapped 95% confidence interval of the median.

Table S4. Free parameters per model

| **Model** | **Global parameters** | **Parameters per condition** |
| --- | --- | --- |
| CNI  CNI priors  CNI priors and risk attitude | *τ*, *k_0_*  *τ*, *k_0_, α*_0_*,* *β*_0_  *τ*, *k_0_*_,_ *α*_0_*,* *β*_0_, *λ* | *c* (cost and no cost), *ω* (overt cost, overt no cost)  *c* (cost and no cost), *ω* (overt cost, overt no cost)  *c* (cost and no cost), *ω* (overt cost, overt no cost) |
| Threshold_s_  Threshold_a_  Threshold_c_ | *τ*  *τ*  *τ, s* | *b*  *b*_+_, *b*_-_  *b* |
| Sample cost  Sample cost priors  Sample cost priors, risk attitude | *τ*, *k_0_*  *τ*, *k_0_, α*_0_*,* *β*_0_  *τ*, *k_0_*_,_ *α*_0_*,* *β*_0_, *λ* | *c*  *c*  *c* |
| Uncertainty  Uncertainty priors | *τ*  *τ, α*_0_*, β*_0_ | *k*  *k* |

*τ =* Decision noise temperature, = *k*_0_= softmax intercept, *λ* = risk attitude*, α*_0_ = alpha prior*, β*_0_ = beta prior, *c* = sampling cost, *k =* stopping criterion, *s* = speed of bound collapse, *b* = decision bound.

Through parameter recovery, we verified that the number of trials was large enough to accurately estimate parameters. We used the parameter estimates of 20 randomly selected subjects to simulate data for parameter recovery. This was done to ensure that the simulated data reflected the level of decision noise that was present in the participants data. The data was simulated with an equal number of trials as in the actual task. We then fitted that simulated data to the model to check if the parameters values that generated the data were correctly estimated. We subtracted the estimated parameters of the simulation from the actual input parameters and calculated the bootstrapped 95% confidence interval and found no significant difference between the input parameters and the recovered parameter values.

To test if the model predictions could be distinguished from each other and test for biases against any particular model, we performed model recovery. We used the parameter estimates to generate data from each model, and fitted those data to each model. This shows that all three models were recoverable, as data generated by a model was also best fitted by that model (Table S5).

Table S5. Model recovery results

|  | | | **AIC** | | | | **BIC** | | |
| --- | --- | --- | --- | --- | --- | --- | --- | --- | --- |
|  |  | | | 95% CI | |  | | 95% CI | |
|  | | sum | | lower | upper | median | | lower | upper |
| **Data generated by the CNI model** | |  | |  |  |  | |  |  |
| CNI vs. Sample cost | | -1947 | | -2514 | -1396 | -1947 | | -2585 | -1367 |
| CNI vs. Uncertainty | | -2909 | | -3473 | -2400 | -2679 | | -3233 | -2199 |
| CNI vs. Threshold | | -2145 | | -3559 | -639 | -1685 | | -3185 | -319 |
| **Data generated by the Sample cost model** | |  | |  |  |  | |  |  |
| Sample cost vs. CNI | | -388 | | -702 | -54 | -388 | | -738 | -109 |
| Sample cost vs. Uncertainty | | -2758 | | -3176 | -2175 | -2516 | | -3060 | -1980 |
| Sample cost vs. Threshold | | -3833 | | -4673 | -2972 | -3349 | | -4273 | -2466 |
| **Data generated by the Uncertainty model** | |  | |  |  |  | |  |  |
| Uncertainty vs. CNI | | -2681 | | -3634 | -1709 | -2912 | | -3888 | -2018 |
| Uncertainty vs. Sample cost | | -2581 | | -3710 | -1657 | -2811 | | -3740 | -1978 |
| Uncertainty vs. Threshold | | -3210 | | -4187 | -2474 | -2402 | | -3356 | -1674 |
| **Data generated by the Threshold model** | |  | |  |  |  | |  |  |
| Threshold vs. CNI | | -1168 | | -1658 | -703 | -2201 | | -2754 | -1667 |
| Threshold vs. Sample cost | | -1654 | | -2140 | -1165 | -2114 | | -2641 | -1612 |
| Threshold vs. Uncertainty | | -1723 | | -2383 | -1164 | -1953 | | -2523 | -1424 |

95% CI = Bootstrapped 95% confidence interval of the summed difference between model fits. Negative values indicate a better fit of the model that generated the data. The data were generated using the participants’ parameter estimates. This shows that all models were recoverable.

***Study 2***

Each subject completed a total of 200 trials (40 trials per *r*), resulting in a maximum of 1000 sampling decisions per probability. There were 42 subjects in the positively biased condition, the data from 3 participants were excluded because they reported confusion about the task, resulting in 39 subjects (14 males, age: *m* = 23.15, *sd* = 2.75, range = 18-34 years). There were 39 subjects in the negatively biased condition, data from 2 participants were excluded because they did not understand the instructions, and data from 1 participant was excluded due to a technical malfunction, resulting in 36 subjects (11 males, age: *m* =21.16, *sd* = 3.13 range = 18-34 years). A one-way ANOVA showed no significant age difference between any of the groups in Study 1 and Study 2 (*p* = .091).

*Descriptive statistics Study 2*

We conducted a mixed-effects model on the log transformed number of samples. The model included the interaction between monetary cost and social context, and the interaction between bias, outcome uncertainty, and reciprocation probability. This replicated all results of the analyses in study 1. However, further examining the interaction effect between social context and monetary cost, we found that the effect of social context was present when sampling was free (*β =* 0.200±0.016, *p* < 0.001), but was not significant when it was costly (*β =* 0.020±0.016, *p* = 0.225).

Table S6. Mixed-effects model results study 2

|  | **logSamples** | | | |
| --- | --- | --- | --- | --- |
| *Predictors* | *Estimates* | *95%CI* | *Statistic* | *p* |
| (Intercept) | 2.49 | 2.26 – 2.71 | 21.56 | **<0.001** |
| MonetaryCost | -0.76 | -0.79 – -0.72 | -46.50 | **<0.001** |
| SocialContext | -0.20 | -0.23 – -0.17 | -12.15 | **<0.001** |
| Bias | 0.02 | -0.24 – 0.29 | 0.15 | 0.879 |
| Uncertainty | 0.82 | -0.27 – 1.92 | 1.47 | 0.141 |
| ReciprocationProb | -0.42 | -0.66 – -0.18 | -3.46 | **0.001** |
| MonetaryCost *SocialContext | 0.18 | 0.13 – 0.22 | 7.76 | **<0.001** |
| Bias * Uncertainty | 0.11 | -1.03 – 1.25 | 0.19 | 0.850 |
| Bias * ReciprocationProb | -0.37 | -0.74 – 0.00 | -1.96 | 0.050 |
| Uncertainty * ReciprocationProb | 3.18 | 1.54 – 4.82 | 3.79 | **<0.001** |
| (Bias * Uncertainty) * ReciprocationProb | 0.97 | -1.11 – 3.06 | 0.91 | 0.361 |
| **Random Effects** | | | | |
| σ^2^ | 0.29 | | | |
| τ_00_ _subjects_ | 0.19 | | | |
| ICC | 0.39 | | | |
| N _subjects_ | 78 | | | |
| Observations | 8787 | | | |
| Marginal R^2^ / Conditional R^2^ | 0.225 / 0.529 | | | |

We then used a separate logistic regression to test whether the decision to invest was predicted by *r* and the cost conditions. This also replicated our findings, as the regression returned 2.989±0.048, *p* < 0.001 for *r*, indicating that the probability of investing increased with a higher *r*. This confirms that the acquired information was actually used in the final investment decision. As expected, the two cost manipulations (monetary and social) and their interaction were not significant predictors of the decision to invest (monetary cost: -0.031±0.027, *p* = 0.248; social context: -0.045±0.027, *p* = 0.091; interaction between monetary and social context: 0.047±0.027, *p* > 0.081).

Table S7. Model comparisons Study 2

|  | | | **AIC** | | | | **BIC** | | |
| --- | --- | --- | --- | --- | --- | --- | --- | --- | --- |
|  |  | | | 95% CI | |  | | 95% CI | |
|  | | sum | | lower | upper | sum | | lower | upper |
| **Within-models comparisons** | |  | |  |  |  | |  |  |
| CNI basic - priors | | 3273 | | 2678 | 3899 | 2409 | | 1812 | 3033 |
| CNI priors – risk attitude | | 2385 | | 1515 | 3407 | 1953 | | 1082 | 2982 |
| Sample cost basic – priors | | 1755 | | 1184 | 2367 | 891 | | 322 | 1504 |
| Sample cost priors - risk attitude | | 5442 | | 4142 | 6862 | 5010 | | 3714 | 6426 |
| Uncertainty basic - priors | | 6145 | | 3925 | 8855 | 5281 | | 3072 | 7983 |
| Threshold_s_ – Threshold_a_ | | 6561 | | 3388 | 9684 | 4833 | | 1657 | 7977 |
| Threshold_a_ – Threshold_c_ | | -3853 | | -9244 | -870 | -2557 | | -7944 | 422 |
| **Between-models comparisons** | |  | |  |  |  | |  |  |
| CNI vs. Sample cost | | 529 | | -128 | 1450 | 529 | | -128 | 1450 |
| CNI vs. Uncertainty | | 2304 | | 738 | 3891 | 3168 | | 1598 | 4759 |
| CNI vs. Threshold | | -4072 | | -5352 | -2801 | -2345 | | -3630 | -1067 |
| Sample cost vs. Uncertainty | | 1776 | | -128 | 3594 | 2638 | | 734 | 4462 |
| Sample cost vs. Threshold | | -4600 | | -6226 | -3145 | -2873 | | -4505 | -1410 |
| Uncertainty vs. Threshold | | -13538 | | -16893 | -10195 | -12674 | | -16032 | -9332 |

95% CI = Bootstrapped 95% confidence interval of the summed difference between model fits. Because of the summation of the difference, large positive or negative numbers therefore reflect that one model wins consistently, i.e. for most subjects. Lower AIC and BIC values indicate a better model fit. Thus, positive values indicate a better fit for the second model, while negative values indicate a better fit for the first model. The models were fitted using an identical procedure to that described in study 1. The distribution of generative reciprocation probabilities was biased in study 2. For the basic CNI, Sample cost, and Uncertainty models, we therefore fixed the priors based on the true distribution (alpha = 0.84 and beta = 0.56) for the positively biased distribution and vice versa for negatively biased distribution.

Table S8. Median estimated condition parameters for heuristic models Study 2

| **Positive Bias** | | Trustee not informed | | | Informed | | |
| --- | --- | --- | --- | --- | --- | --- | --- |
|  |  | | 95% CI | |  | 95% CI | |
|  | | median | lower | upper | median | lower | upper |
| Uncertainty model *k* monetarily free | | 0.052 | 0.030 | 0.061 | 0.062 | 0.048 | 0.073 |
| Uncertainty model *k* monetarily costly | | 0.100 | 0.084 | 0.112 | 0.101 | 0.089 | 0.113 |

| **Negative bias** | |  | | | |  | | |
| --- | --- | --- | --- | --- | --- | --- | --- | --- |
| Uncertainty model *k* monetarily free | 0.014 | | 0.00 | 0.028 | 0.057 | | 0.023 | 0.074 |
| Uncertainty model *k* monetarily costly | 0.108 | | 0.094 | 0.114 | 0.106 | | 0.094 | 0.125 |

95% CI = Bootstrapped 95% confidence interval of the median.

*Subjective reports Study 2*

The subjective reports in study 2 replicated the pattern in study 1: Over half of the participants believed that the reciprocation probability *r* would decrease when information was overtly sampled (51% and 59% in positive and negative bias conditions, respectively). Fewer believed that the probability would become larger (27% and 15% in the positive and negative bias conditions, respectively). The remaining participants believed it would not affect *r*.

**References**

1. Bates, D., Mächler, M., Bolker, B. & Walker, S. Fitting linear mixed-effects models using lme4. *arXiv preprint arXiv:1406.5823* (2014).

2. Kuznetsova, A., Brockhoff, P. B. & Christensen, R. H. B. lmerTest package: tests in linear mixed effects models. *Journal of statistical software* **82**, (2017).

3. Bellman, R. On the theory of dynamic programming. *Proceedings of the National Academy of Sciences of the United States of America* **38**, 716 (1952).

4. Drugowitsch, J., Moreno-Bote, R., Churchland, A. K., Shadlen, M. N. & Pouget, A. The cost of accumulating evidence in perceptual decision making. *Journal of Neuroscience* **32**, 3612–3628 (2012).

5. Skowronski, J. J. & Carlston, D. E. Negativity and extremity biases in impression formation: A review of explanations. *Psychological bulletin* **105**, 131 (1989).

6. Siegel, J. Z., Mathys, C., Rutledge, R. B. & Crockett, M. J. Beliefs about bad people are volatile. *Nature human behaviour* **2**, 750–756 (2018).

7. Baumeister, R. F., Bratslavsky, E., Finkenauer, C. & Vohs, K. D. Bad is stronger than good. *Review of general psychology* **5**, 323–370 (2001).

8. Tajima, S., Drugowitsch, J. & Pouget, A. Optimal policy for value-based decision-making. *Nature communications* **7**, 12400 (2016).
